# Global expression profiling in leaves of free-growing aspen

**DOI:** 10.1186/1471-2229-8-61

**Published:** 2008-05-23

**Authors:** Andreas Sjödin, Kirsten Wissel, Max Bylesjö, Johan Trygg, Stefan Jansson

**Affiliations:** 1 Um eå Plant Science Centre, Department of Plant Physiology, Um eå University, SE-901 87 Um eå, Sweden; 2 Research Group for Chemometrics, Department of Chemistry, Um eå University, SE-901 87 Um eå, Sweden; 3 Department of Otolaryngology, Medical University of Hannover, Carl-Neuberg Str. 1, D-30625 Hannover, Germany

## Abstract

**Background:**

Genomic studies are routinely performed on young plants in controlled environments which is very different from natural conditions. In reality plants in temperate countries are exposed to large fluctuations in environmental conditions, in the case of perennials over several years. We have studied gene expression in leaves of a free-growing aspen (*Populus tremula*) throughout multiple growing seasons

**Results:**

We show that gene expression during the first month of leaf development was largely determined by a developmental program although leaf expansion, chlorophyll accumulation and the speed of progression through this program was regulated by the temperature. We were also able to define "transcriptional signatures" for four different substages of leaf development. In mature leaves, weather factors were important for gene regulation.

**Conclusion:**

This study shows that multivariate methods together with high throughput transcriptional methods in the field can provide additional, novel information as to plant status under changing environmental conditions that is impossible to mimic in laboratory conditions. We have generated a dataset that could be used to e.g. identify marker genes for certain developmental stages or treatments, as well as to assess natural variation in gene expression.

## Background

The plant leaf is a fantastic organ that provides the plant, and ultimately mankind, with carbohydrates that are essential for growth and maintenance. In many species leaf morphology is remarkably plastic, the shape and size of leaves that develop under diverse conditions may differ substantially both in plants of the same species and even on individual plants [1]. Leaf differentiation is controlled by a genetic program, but to allow for plasticity in leaf shape, the developmental program is modified by environmental factors [2]. After the cell expansion phase, secondary cell wall formation occurs and the cell is subsequently unable to change its shape. However, developmental programs continue to modify gene expression in the leaf in adaptive responses that optimize its capacity to perform its main function, photosynthesis.

In most plants leaves are formed from apical meristems throughout the course of their vegetative development and leaves at various developmental stages may often be present. Most trees also have an indeterminate growth pattern, forming leaves throughout the growing season and leaf plastochron indices (LPIs) can sometimes be used to describe the different leaf developmental stages along the stem [3]. Some trees, however have determinate growth, whereby all leaves for the next growing season are formed but developmentally arrested in winter buds that will flush in a synchronized manner the following spring. Leaf development from winter buds of trees is a somewhat unusual developmental pathway. In the bud, leaf development is arrested at an early stage, internode elongation is inhibited and, if the tree has a determinate growth pattern, most or all leaves that will develop in the coming season overwinter in the bud. When buds are reactivated in the spring, most leaf expansion is thought to be result from cell expansion, but cell division also occurs and the temporally overlapping cell division and expansion determine the final size and shape of the leaves [4,5]. Young aspen trees typically have an indeterminate growth pattern and produce new leaves until the critical day length has been reached, when growth arrest and bud set occur. Fully-grown aspens, and many other tree species, in Sweden (where the growing season is short) have however only one flush, so at a given date all leaves are of identical age.

The interaction between environmental and developmental factors that influence leaf development and gene expression can be addressed in several ways, for example by analysing plants with mutations that affect leaf development [6] or by exposing plants to highly controlled conditions and measuring changes in gene expression or developmental parameters following changes in specific variables [7]. A third strategy that has been much less frequently employed is to monitor leaf development and gene expression under natural, uncontrolled conditions [8]. There are considerable challenges in such an approach. For example, stochastic changes in weather parameters or biotic interactions may obscure the main intrinsic processes regulating leaf gene expression and development, and when responses are measured bias in the selection of the measured parameters may influence the results. In attempts to generate interpretable data regarding the complex phenomena and highly interacting factors involved, we are combining several scientific approaches, including high throughput genomic analyses to measure gene expression (or at least mRNA levels), together with morphological, physiological and ecological evaluations of plants grown under natural conditions. We have shown that multivariate statistical treatment of gene expression data from leaves of an aspen tree grown under uncontrolled, highly variable, conditions can be analyzed to separate developmental, or rather leaf age-dependent, factors from environmental factors influencing gene expression [8]. This prompted us to embark on a project to analyse the global pattern of gene regulation in leaves of a tree grown under natural conditions in the most efficient way, to complement data on gene expression in leaves in the model annual plant *Arabidopsis *[9,10].

Instead of running DNA microarrays on samples from every day, we believe that a strict sample selection scheme, guided by experimental design and multivariate statistics, aiming at maximizing the information content in the results, can be used. Considering that our experimental tree has determinate growth and based on our previous studies, the life span of an aspen leaf could be divided into three phases. Early in the season (in Um eå from late May up to late June) leaves develop from buds, reach maturity and at the end of the season they senesce. In between, there is a time window when the leaf is neither growing nor senescing. Based on expression data from eleven genes, we have shown that the changes in gene expression are larger in early and late season, whereas in the middle season, gene expression is more constant [8]. When the leaf is not growing, but has not yet started to senesce, gene expression is, in theory, only needed 1) if the protein composition of the leaf should be changed due to changes in environmental conditions or 2) to replace proteins that have been degraded. Therefore this time period is maximally informative to analyse environmental influences on gene expression.

This study aims to answer several questions about gene regulation in aspen leaves. For example, can DNA microarrays best be used to get high-quality expression data to faithfully monitor the biosynthetic activities in leaves of a tree growing under natural, uncontrolled conditions? Which weather factors are most important for determining mRNA levels? How similar, or different, is gene expression in the same individual if several seasons are compared? To do so we have constructed a timetable for gene expression during the entire course of aspen leaf development from initiation through to senescence and have pinpointed environmental effects during different phases of the growing season.

## Results

### The developmental pattern in gene expression over the season could be visualized using DNA microarrays

**Figure 1:**
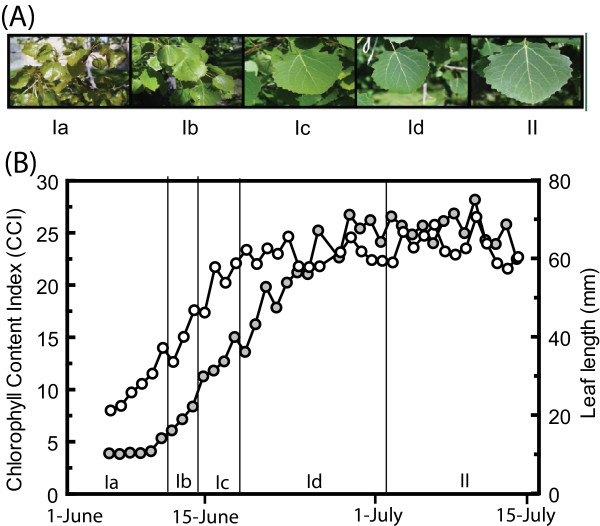
**Correlation between chlorophyll and leaf length**. Leaf development from Phase I a-d and Phase II illustrated (A) by photographs of leaves and (B) measurements of leaf length (white circles) and chlorophyll content indices (grey circles).

We first wanted to gain a physiological overview of leaf development, which we defined using leaf size (expansion) and chlorophyll accumulation. This showed that chlorophyll accumulation lagged behind the onset of expansion by one week but then continued for another 2 weeks after leaves had stopped expanding (Figure 1). Bud burst in trees can be accurately modelled by calculating the temperature sum or 'day-degrees' above a certain threshold temperature (typically +1°C to +5°C), after the release from dormancy [11]. We wanted to see if temperature also regulated leaf expansion and chlorophyll accumulation in leaves of aspen. It is not known exactly when release from endodormancy occurs in aspens, but it is certainly long before the end of March (Rishikesh Bhalerao, pers. comm.). If leaf length was plotted against temperature sum, using a threshold temperature of +1°C, there was an almost perfect linear fit between leaf length and temperature sum (R^2 ^= 0.97), illustrating the temperature dependence of leaf expansion up to June 19, when the temperature sum reached 500 (data not shown). Moreover, if chlorophyll levels were compared with temperature sum, again an almost perfect fit was found, up to June 28, corresponding to temperature sum 700. Thus, temperature appeared to be the major determinant of leaf expansion and chlorophyll accumulation from bud burst up to the stage when the leaf was fully developed.

In the first part of this project, we focused on genes primarily regulated by leaf age/developmental stage. We had sampled leaves from our experimental tree every day for several years, and could therefore choose among many different leaf samples for analysis with microarrays. Most informative in this respect would be samples where all weather parameters were as similar as possible, whereas the difference in developmental stage would be as large as possible. Therefore, we wanted to select sampling days spread throughout the growing season with similar weather conditions. To do so in a statistically stringent way, we applied Principal Component Analysis (PCA) to weather data recorded by a weather station about 200 m from the studied tree (for details see Material and methods). An overview of the weather conditions throughout the growing season in the year 2000 have been published previously [8]. From the PCA representation of the calculated weather parameters for each day [See Additional file 1 and 2], we selected ten days evenly spread over the growing season. The weather on the selected days was sunny with maximum day temperatures between 10 and 20°C. We also included an additional sample (July 18) [see Additional file 3], in the middle of the season, in which the weather was significantly different (rainy).

**Figure 2:**
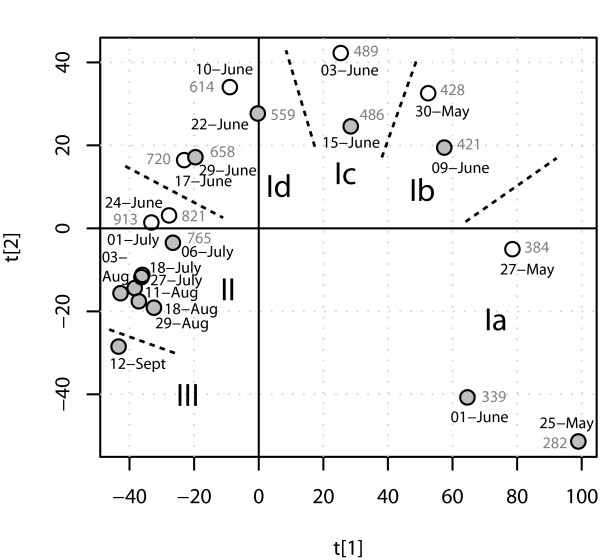
**Overview of gene expression in leaves of free-growing aspen during the growing season**. Comparison of gene expression in leaves of free-growing aspen during the growing season of 2000 (grey circles) and 2002 (white circles). The first two principal components obtained from the PCA analysis of the microarray data are shown. Temperature sums (day degrees, see Material and methods) for the days of sampling are indicated, while Ia, Ib, Ic, Id, II and III corresponds to gene expression sub-phases, described in the text

We analyzed gene expression in the eleven selected dates, using DNA microarrays, in four replicates against a common reference. Slides with poor quality due to uneven hybridization were removed from further analysis. We then applied PCA to obtain an overview of the dataset and, in an unsupervised manner, summarize the variation in gene expression in the samples (Figure 2). The eleven samples from the whole growing season showed a very clear developmental trend, as illustrated by the PCA score plot [see Additional file 4]. The fact that replicated samples clustered closely together illustrate the high reproducibility of our array analysis. A time line could be drawn through the diagram, in which the samples appeared in a chronological order. This shows the success of the approach we used to select samples for the experiment, since noise in the gene expression profiles due to stochastic environmental factors was largely eliminated, allowing the effects of the developmental program to be clearly distinguished.

Most of the changes in developmentally regulated gene expression appeared to occur before July 1, when leaves were expanding and internal structures are formed. These observations prompted us to analyze gene expression in more detail, in order to improve the temporal resolution of the analysis, during this phase. For this reason we included in the experimental setup three additional samples for analysis during the same time period. In addition, we decided to also include another seven samples from the same time period, but from another year (2002). This year was chosen since the weather during this period in 2002 was unusually warm, while May and June 2000 were unusually cold, and it could therefore inform about how temperature influenced gene expression during the time of leaf development.

We analyzed all these arrays in the same way as mentioned above. An overview of the total pattern of gene expression over two seasons confirms that the major changes in gene expression occurred during the first month after bud burst. The first dimension (t1) of the PCA score plot (Figure 2), which describes most (37%) of the variation, separates the samples from May and June from the remaining samples, showing that the main differences in gene expression occurred before July 6 2000 and 1 July 2002. The second dimension (t2), which describes 15% of the variation, sorts primarily the rest of the samples in chronological order, and clearly separates the July 6 and September 12 samples from the others. However, the samples from August 3 to August 29 were not convincingly separated from one another in either the first or second dimension. Apparently, during July and August, leaf-age dependent changes in gene expression were minor, and there were no clear developmental trends in gene expression, especially during the period around August 1 (Figure 2). As the season progressed, larger changes in gene expression occurred and the expression profile of the sample from September 12 was quite different from that of all preceding samples. This coincides, of course, with early stages of the autumn senescence process, although chlorophyll degradation has barely started by this date, and visible leaf senescence does not occur until long after it [12,13]. The sample from July 18, when the weather was different, did not stand out as an obvious outlier.

### Temperature determines the rate of progression through the leaf developmental program

Inclusion of samples from two seasons not only increases the resolution of the analysis at these growth stages, but also provides additional information concerning the relationship between development and environmental factors, since the weather in June 2000 and June 2002 was so different. However, the transcriptomes of all samples could by large be connected with a single curve, implying that although the leaves were exposed to different weather conditions in 2000 and 2002, they went through basically the same 'linear' developmental program, i.e. patterns of gene expression changed in a predetermined way that was only to a minor extent influenced by the weather conditions. However, the leaves clearly went through this program at different rates in the two years. In 2000 the program started later (i.e. bud burst was later) and the leaves progressed along the developmental axis much more slowly than in 2002; May 27 2002 corresponded to June 3 or 4 in 2000, whereas the gene expression profile on June 3 of 2002 was similar to that of June 16 in 2000 (Figure 2). We therefore wanted to find out whether or not the state of the transciptome, not only leaf expansion and chlorophyll accumulation, was also determined by the temperature sum. We calculated temperature sums from January 1 for the years 2000 and 2002. The cumulative sum of day degrees, indicated in Figure 2, showed that days in the 'transcriptome state' correlated almost perfectly with the temperature sum, consistent with the hypothesis that not only bud burst, leaf expansion and chlorophyll accumulation but the whole process of leaf development, from bud burst until the leaves were mature, depended on the temperature sum.

Based on these findings, we subdivided the 'leaf development period' e.g. the time period covered by the array experiments for the two years (Phase I) into sub-phases. We assigned the samples from May 25 2000, June 1 2000 and May 27 2002 to Phase Ia (temperature sum 250–400), the samples from June 9 2000 and May 30 2002 to Phase Ib (temperature sum 400–450), from June 15 2000 and June 3 2002 to Phase Ic (temperature sum 450–500) and from June 22 2000, June 10 2002, June 29 2000 and June 17 2002 to Phase Id (temperature sum 500–750). Since the leaves entered Phase II ('mature leaves') after June 29 2000, the samples from June 24 2002 and July 1 2002 were assigned to Phase II. The transition from Phase Ia to Ib coincided with the start of chlorophyll accumulation, and thus chloroplast development, in the leaves and the transition from Phase Ic to Id corresponded to when leaves stopped elongating (Figure 1). The temperature sum that appeared to be necessary for the leaf to complete Phase I and enter Phase II was therefore around 350. The whole process, from the first comparable transcriptional stage (late Phase Ia, May 27 2002 comparable to June 3–4 2000) to Phase II took about 25 days in 2000 but only 14 days in 2002, illustrating the strong impact of temperature on the role of leaf development.

### Gene expression during leaf development reflected key cellular events

**Figure 3:**
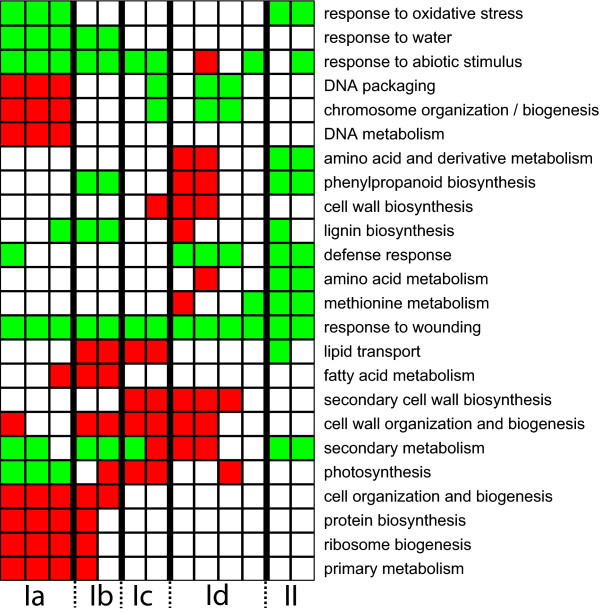
**Heatmap of the over-represented Gene Ontology categories in the spring samples**. The samples from the spring 2000 and 2002 are ordered according to developmental leaf age. Red squares are categories over-represented in significantly up-regulated genes and green squares are categories over-represented in significantly down-regulated genes. Divergent groups that were over-represented in both up- and down-regulated genes are plotted as yellow squares. This figure shows selected categories of interest, for the full image please see Additional file 5.

Translating the information in expression data to biologically relevant information is a major task for biologists engaged in transcriptomic analyses. Having defined a number of leaf transcriptome phases and placed them on two year's calendars, we aimed to describe the transcriptional activities at each of the phases, to allow us to draw conclusions about the main cellular activities at each developmental stage. The microarray technique still gives rather noisy data and studies of groups of genes in appropriate functional classes gives much more robust data than analyses of individual genes [14]. Therefore, we ordered the samples from year 2000 and 2002 according to their respective developmental stage, as described above, and examined the gene expression patterns in the dataset, which can be viewed in UPSC-BASE [15]. In order to determine, in an unsupervised manner, the main metabolic activities during each of the Phase I sub-phases we classified the array elements on the *Populus *microarrays according to the Gene Ontology (GO) classifications of the closest *Arabidopsis *orthologues. We subsequently identified the GO classes, using the GOstats package in Bioconductor [16], that were significantly over-represented in the sets of up-or down-regulated genes in each of the samples, ordered according to temperature sum/leaf developmental stage. In Figure 3 and Additional file 5, GO categories that were over-represented in up-regulated genes are colored red, while those with significant over-representation in down-regulated genes are colored green. During Phase Ia leaves are expanding but chlorophyll accumulation has not yet started (Figure 1). The major transcriptional pattern during Phase Ia is a heavy up-regulation of genes coding for ribosomal proteins (e.g. genes in the Protein biosynthesis and Ribosome biogenesis classes) and histones representing classes, such as Chromatin assembly and chromosome organization. Other up-regulated GO classes include Cell organization and microtubule-based processes, and DNA metabolism. Expression of many genes with a role in the cell proliferation or pattern formation, such as Cyclin dependent kinase (PttCDKB2) (PU00348) [17], YABBY-like transcription factor (PU09301, PU09931, PU09586, PU08954, PU26269, PU09515) [18], retinoblastoma (PU09599, PU01146) [19] and Aintegumenta (PU04170, PU05886, PU02438) [20,21] were also strong during this phase. Many classes of genes were expressed at very low levels in Phase Ia, including genes related to photosynthesis, secondary metabolism, and responses to many biotic and abiotic stimuli such as light/radiation, oxidative stress, water, pathogens, wounding etc. Genes involved in ethylene biosynthesis were weakly expressed in Late Phase Ia and early Ib. During Phase Ia, the leaf is rapidly growing and this growth is apparently accompanied by significant cell division activity whereas many of the metabolic pathways typical of leaves, such as photosynthesis, secondary metabolism and stress responses, are kept on hold.

In Phase Ib, the GO classes that are typically highly expressed in Phase Ia (Ribosomal proteins, Histones, DNA metabolism etc.) are not over-represented. Thus, cell division activities appear to occur mainly in a short period of time in aspen leaves developing from winter buds. However, the expression levels of genes encoding components of the photosynthetic apparatus and chlorophyll biosynthesis increased strongly. Several categories of proteins related to lipid metabolism (Fatty acid biosynthesis, Lipid transport, Lipid biosynthesis) were also up-regulated in Phase Ib. The categories Carbohydrate metabolism, Cell wall organization and Biogenesis were also over-represented in Phase Ib, consistent with Phase Ib being a phase where cell elongation mainly occurred. Classes with particularly low expression in Phase Ib included Lignin biosynthesis, indicating that secondary cell wall formation had not yet started. Clearly, cells were rapidly elongating, the primary cell wall was synthesized and the proplastids were developing into chloroplasts during this phase. At the individual gene level, tubulins were up-regulated throughout both Phase Ia and Ib. The expression of several genes involved in cell wall biosynthesis peaked in these sub-phases, including genes encoding expansin (exp1, in contrast to other expansin genes, for which expression levels were highest in Phase Id), arabinogalactan proteins AGP18, AGP20 FLA10, proline-rich protein (PRP4), pectin methylesterase-like protein, a germin like protein (GER3) and pectate lyase. A cellulose synthase began to be highly expressed in Phase Ib and its expression level remained high up to the beginning of phase II. The high expression of genes coding for photosynthetic proteins and enzymes in pigment biosynthesis also continued in Phases Ic and Id. In addition, categories such as Lipid transport and Cell wall organization and biogenesis were highly expressed. Overall, there were considerable similarities between the Phase Ib and Ic transcriptomes, but some categories differed. For example, phenylpropanoid and lignin biosynthesis was significantly under-represented in Phase Ib, but not in Phase Ic. GO classes overrepresented in Ib, but not in Ic, included lipid and fatty acid biosynthesis and cellular morphogenesis. This suggests that some, but not many, cells in Phase Ic had stopped elongating and started secondary cell wall biosynthesis. In the last sub phase of Phase I (Id) cells had stopped elongating (Figure 1) and genes coding for enzymes involved in lignin biosynthesis were highly expressed. In addition, classes related to amino acid – in particular methionine – biosynthesis were over-represented, as was the class Carbohydrate metabolism.

Sucrose synthase, which was highly expressed in Phase Ia, but weakly in Ib and Ic, was most strongly expressed during sub-phase Id. During this sub-phase, primary cell wall biosynthesis has largely ceased and secondary cell walls are formed. The completion of secondary cell wall biosynthesis and the concomitant down-regulation of genes involved in lignin biosynthesis marked the end of Phase I and entry into Phase II. When chlorophyll no longer accumulated and the apparently leaf was fully mature. It should also be noted that many categories were under-represented throughout Phase 1, including responses to a wide range of abiotic and biotic stresses, as well as secondary metabolism.

### Weather-regulated gene expression

Secondly, we wanted to focus on environmental influence of gene expression. Although weather, at least temperature, influenced the speed of progression of the transcriptional program also in young leaves, the best resolution in the analysis of environmental influence on gene expression could be achieved in mature leaves when leaf-age dependent changes were minimal. Based on the weather data from the different years, we looked for a time period when the weather conditions were least stable. The time period from August 7 until August 16 had – especially in 2003 but also in 2004 – variable weather and was in the period when leaf-age dependent changes in gene expression by large were absent so environmental signals should be the major inducers and suppressors of gene expression. We selected 10 leaf samples harvested from these time periods and analyzed their gene expression using the DNA microarrays. These data were analyzed with Orthogonal projections to latent structures (OPLS), a supervised multivariate linear regression method [22]. In relation to the unsupervised PCA method, OPLS requires and makes use of additional background information; for instance presence of different sample groups (classes) [23] or treatment concentrations. The special property of OPLS in this context is the ability to separately describe information in the data table that is related to the modelling aim (e.g. to discriminate between different classes) and other systematic trends. OPLS was used to predict gene expression levels based on a description of the current and previous weather, including sunlight, temperature, humidity, wind and precipitation parameters [see Additional file 1 and 2]. Cross-validation [24] was performed to find an OPLS model with good generalization properties. Based on properties of the cross-validated OPLS model, microarray elements that were reliably predicted from the weather parameters were subsequently identified, suggesting that such elements are influenced by or directly regulated by the weather.

**Figure 4:**
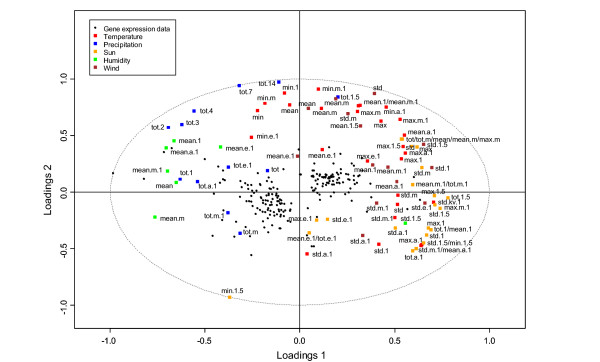
**Biplot of the weather parameters and the modelled genes**. The modelled genes are represented as black dots and the weather parameters are coloured according to the legend. In addition to the colour each parameter is label with the calculation (min = minimum, max = maximum, mean = average, tot = cumulative sum, std = standard deviation) and day or time of measurement (m = morning, a = afternoon, e = evening). A number without additional letter corresponds to cumulative time period. The plot pinpoints the relationship between the different calculated weather parameters used for the modelling of the gene expression. Weather parameters close to each other in the plot are having a similar impact on gene expression. The weather parameters are further described in additional file 1 and 2.

**Figure 5:**
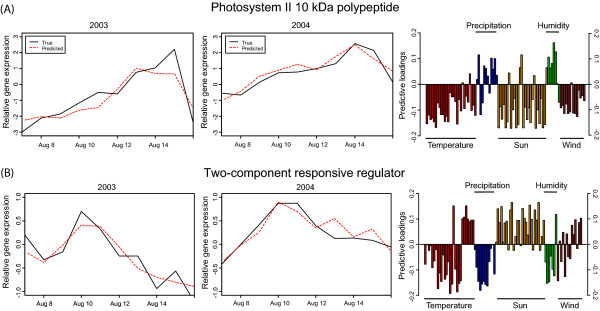
**Characteristic of modelled gene expression**. The two first columns show the relative gene expression (black = measured, red = predicted) and the third column correspond to the positive or negative correlation of the weather parameters relative to the gene expression. The third column describes calculated weather parameters and length of bars corresponds to their impact on the gene expression. The weather parameters are further described in additional file 1 and 2. (A) Photosystem II 10 kDa is negatively correlated with temperature, sun and wind but positively correlated with precipitation and humidity (i.e. had a higher expression on rainy days). (B) two-component responsive regulator is positively correlated with temperature variations and sun but negatively correlated with static temperature and sun (i.e. had a higher expression on when the temperature varying).

In the biplot (Figure 4) the 199 genes with good prediction are shown together with weather parameters. The relation between the different weather parameters is highlighted where parameters close to each other are having a similar impact on gene expression. Weather parameters close to genes are positively correlated with nearby genes while genes opposite in the plot are negatively correlated. Weather parameters far away from origo have stronger influence on included genes. As expected, temperature and sun are strongly correlated while humidity and precipitation show opposite influence. The global OPLS model describes a many-to-many relationship between microarray elements and weather parameters, but OPLS could also be used to pinpoint the weather parameters that are most important for the regulation for a particular microarray element. For this purpose, we constructed local OPLS models for each microarray element where weather parameters were used to sequentially predict one microarray element at a time. From the predictive loadings of each local model, an approximation of the influence of weather parameters for each individual microarray elements is achieved. Two examples of models are shown in Figure 5, where both the relation between measured and predicted mRNA levels is shown, and also the weather parameters that were most correlated with gene expression. Similar to what was shown in Wissel et al, mRNA levels could in these cases be accurately predicted by the weather parameters, and the relative importance, and direction, of the different factors could be estimated. For example, Photosystem II 10 kDa (PsbR) genes mRNA levels were negatively correlated with temperature, sun and wind but positively correlated with precipitation and humidity (i.e. had a higher expression on rainy days) while two-component responsive regulator genes mRNA levels were positively correlated with temperature variations and sun but negatively correlated with static temperature and sun (i.e. had a higher expression on when the temperature varying). PsbR has previously been reported to be under dehydration-rehydration control in *Xerophyta humilis *[25] but contrasting results for *Arabidopsis *[26]. The two-component responsive regulator genes belong to the GARP-ARR-B transcription factor family [27]. The ARR-B family is showing overlapping expression patterns in *Arabidopsis *and might be light-regulated [28].

Finally, we compared the genes differentially regulated in the two sub experiments. The 'cut-off' for being under developmental or environmental control or not is somewhat arbitrary, but using the same criteria in both analyses, – positive B-values [29] – we defined 7615 array elements as having their mRNA levels mainly determined by the stage of leaf development in the early season compared to only 2368 clones under weather control in the second set of experiments. 648 array elements were found in both lists. Genes principally under developmental control early in the season were, consistent with the analysis above, involved in the central anabolic processes of the leaf. Gene Ontology categories high over-represented among these genes included, for example translation, photosynthesis, ribosome biogenesis and assembly, organelle organization and biogenesis, ribonucleoprotein complex biogenesis and assembly, and nucleosome assembly. [see Additional file 6]. In contrast, very few GO categories were consistently affected by weather factors, and those that mainly belonged to small or rather ill-defined categories such as 'system development' or 'dehiscence' [see Additional file 7].

## Conclusion

Can we really understand plant growth, development and acclimation by only studying plants grown under controlled conditions in green-houses and in climate chambers? It is obvious that in many cases highly controlled conditions are necessary and without the possibility to reproduce experiments, plant biology would still be in its infancy. Yet, it is similarly obvious that plants grown in the lab behave very differently from plants grown under natural conditions, where biotic and abiotic factors typically vary considerably, and often unpredictably [30]. The overall aim of this contribution is to see to what extent high throughput studies of gene expression on plants grown under natural conditions can give results that are reproducible and, more importantly, informative. We have set up a DNA microarray analysis pipeline [15] in order to process our cDNA microarrays in a standardized way. Here, we use this pipeline to process many samples from aspen leaves harvested from one single tree over several years, after sample selection aimed at optimizing the information content of the samples, based on previous experiments and consideration of the biology of aspen leaves. The standard strategy in gene cataloguing projects such as AtGenExpress [9] is to grow plants under highly defined conditions in order to minimize the biological variation between samples. This is undoubtedly a useful strategy, but we demonstrate that another strategy, monitoring gene expression in single individuals that are exposed to different environmental conditions over time, can also be used. The life history of a plant sets constraints on the strategies that can be applied, and aspen has several disadvantages compared to *Arabidopsis *in this respect. It also has strengths as a model system, for example its large size and perennial growth habit, and since this kind of study could not have been performed in *Arabidopsis*, the two model systems complement each other [31-33].

We believe that this study shows that a developmental program (in this case leaf development) can faithfully be analyzed using samples from uncontrolled conditions. The transcriptome underwent significant changes during the first weeks of leaf development and during this stage the predefined developmental program of the leaves was much more important than environmental parameters in determining the leaf transcriptome. During this time, leaves expand, cells divide and elongate, chloroplasts are formed and the leaf is finally, in our case after 20–30 days depending on temperature, mature. Uncontrolled environmental factors, such as temperature, of course modulate leaf development significantly [34] and this must occur through the differential regulation of certain genes, but the effect on the overall transcriptome is not large enough to hamper this analysis. The progression through the certain 'sub phases' of Phase I, and the dependence on temperature of its speed, is expected and could of course also have been detected by analyzing plants grown under constant conditions, but we believe that obtaining this information from a tree grown under natural conditions adds to the significance of the findings.

Temperature sum has been shown to be an accurate predictor of bud burst in both conifer and broad-leaf trees [35,36] and many authors have used temperature sum-based analyses to draw important conclusions about bud burst [11,37]. Temperature is also likely to be the major determinant of leaf expansion [38] and chlorophyll accumulation. However, temperature sum has not previously been shown, to our knowledge, to be so tightly connected to the leaf transcriptome all the way up to the stage where the leaf is fully mature, internal structures have been formed and secondary cell wall biosynthesis has been completed. This correlation has to be confirmed by studies of more genotypes and over several years, but we can now identify a well-defined set of marker genes to distinguish the different sub-phases (data not shown), making it possible to monitor the progression efficiently through the developmental stages. Likewise, based on our dataset it is possible to select, for example, genes most suitable to use as 'control genes' to normalize e.g. RT-PCR data or the genes indicative of various environmental stresses, without being influenced by leaf age. Of course, our dataset is not as comprehensive as corresponding datasets from *Arabidopsis *leaves [9,10], but we nevertheless believe it will serve as a useful resource for future studies. For example, by including a large number of additional *Populus *leaf microarray datasets present in UPSC-BASE (e.g. after drought stress, herbivore and pathogen treatments, cold treatments, diurnal cycles etc.) we can now perform meta-analyses and identify genes that have similar transcription responses throughout all experiments, i.e. which constitute a regulon. For each of the regulons, we can using the methods developed here, select the variables that most influence gene expression, keeping in mind that different factors are important in growing, mature and senescing leaves. Another possibility is to, for each regulon, select one or more marker genes that could be analyzed in a large number of samples, for example in different genotypes, to dissect natural variation in gene expression, This variation may be substantial in aspen, considering that the genetic variation at the DNA sequence level is unusually high in comparison not only with most annuals and crop plants, but also in comparison with other trees [39]. Such a toolbox could hopefully be used to address many important questions, not only informing about tree biology but also about plant biology in general. Our dataset could also be used to examine the expression pattern of orthologs that have been shown to regulate different aspects of leaf development in *Arabidopsis *[40,41] or to identify novel genes whose expression patterns indicate that they may play a role in various stages of leaf development. These possibilities were beyond the scope of the work presented here, but since our dataset is publicly available in UPSC-BASE [15], such analyses can now be undertaken.

Changes in gene regulation during leaf development are very strong. This has significance for sampling strategies for plants grown under constant conditions. Unless identical developmental stages are sampled, changes in gene expression induced by various treatments are likely to be masked by the larger effect caused by differences in developmental stage. This is important since many treatments, not only temperature, influence leaf development. This could be illustrated by one of our own experiments, where the 'direct effects' of gene expression by treatment with elevated [CO_2_] were, by large, masked by the apparent changes in leaf developmental stage, despite leaves being sampled with the intention that they should be at the same stage (i.e. had the same plastochron index, PI) [42]. Other published DNA microarray experiments may have suffered from the same problems, adding to the lack of consistency often found between results obtained in different experiments.

Two-colour microarray data provide relative measurements of gene expression, and if many samples are analyzed, like in this study, over- and under-representation means in comparison with the whole dataset. This has to be kept in mind during the analysis of the data: low representation of, for example, stress related genes during Phase I is not an absolute measure but means lower than the 'average sample', and since many samples were from stages when the leaves did not grow and therefore have only a low expression of genes involved in 'building the leaf' and therefore, as a consequence, higher expression of all other types of genes. Therefore, we do not think that our data necessarily indicate that expression of genes involved in resistance against biotic and abiotic stress is not very important during Phase I, rather that stress genes are disappearing in the overload of development genes. This is nothing that is special for a study like this, performed in the field, but something that needs to be kept in minds during e g all time series analysis of microarray data.

It should be pointed out that the analysis we have performed here only provides indications regarding the 'average transcriptome' of leaf cells. Clearly, different cell types will have very different gene expression patterns [43,44], and different areas of the leaf will not be in exactly the same developmental phase [45-47]. We believe that cellular development is likely to be more highly synchronized during bud flush than during 'normal' leaf development. This is because many cells may be arrested in the same developmental stage in winter buds, and when conditions are permissive for bud flush, the cellular activities of many cells are synchronized. In continuously growing plants cells in the different parts of the leaf are in different stages, so the overall gene expression profile in the leaf may be more of a blend of many developmental stages, but this needs to be experimentally verified.

Finally, we believe that this contribution also demonstrates some of the power of multivariate statistics in the analysis of gene expression, both in terms of analyzing patterns in DNA microarray data but also to identify breakpoints in e.g. time series, when subtle changes in gene expression characteristics can be pinpointed. Aspen has several disadvantages compared to *Arabidopsis *as model system, but it also has strengths such as large size and perennial growth habit. Another obvious difference between the model systems is the amount of genetic variation. Aspen individuals in a local population have about 1% nucleotide diversity inside genes [39], i.e. two aspens trees are on DNA level more divergent than a human and a chimpanzee. We believe that this is a further argument in favour for our experimental strategy, to do detailed transcript profiling of one single tree, since using different aspen trees as biological replicates would result in lack of resolution in the results similar to the situation if a chimpanzee should be used as a biological replicate for array studies on humans. Our aim is to use this dataset to further look at natural variation in gene expression in aspen individuals, but that is outside the scope of this publication.

## Methods

### Sampling

Leaves were harvested following a controlled procedure from branches 2 m above ground on one single fully grown (>30 years) free-growing aspen tree (*Populus tremula*) on the Umeå University campus (63° 49'17"N, 20° 18'40"E) at 11.00 am on each sampling occasion throughout the entire growing seasons of 2000–2005. The sampled leaves were flash frozen in liquid nitrogen and stored at -70°C awaiting analysis. At least 20 leaves from each occasion were then crushed in liquid nitrogen and mixed. Each replicate used a fraction of leaf mixture for RNA extraction. The samples from 2000 were the same as those used in a previous study [8].

Relevant weather parameters (temperature, humidity, air pressure, wind, sun intensity and precipitation) were calculated from data collected by a weather station located on the Umeå university campus about 200 m from the tree from which the samples were harvested. The data were collected hourly throughout the year and transformed to new parameters describing stability, variation and time lags of original weather parameters. The lagged variables consist of morning, afternoon, evenings and day summaries in different combinations. The calculated lagging variables correspond to environmental factors that develop slowly and not directly measured by the weather station, such as soil water potential and temperature. For more details about the analysis of the weather data see the principal component analysis (PCA) section [24]. Leaf length of 6 leaves were measured with a ruler each day and leaf chlorophyll content was measured with a chlorophyll content meter (CCM-200, Opti-Sciences, Tyngsboro, MA, USA) on the same leaves during the beginning of the growing season 2005.

**Figure 6:**
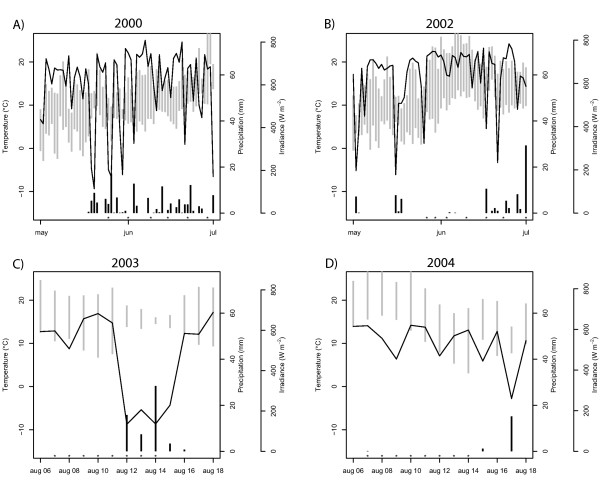
**Overview of weather conditions**. Weather conditions during the detailed spring and weather experiment. The line represents maximum irradiance of the day (W m^-2^), black bars show the daily precipitation (mm) and grey bars denote the maximum and minimum temperature (°C) of the day.

For the first set of microarray experiments, samples collected on eleven days with similar weather conditions were selected (June 1, June 15, June 29, July 6, July 18, July 27, August 3, August 11, August 18, August 29, September 12). Subsequently, further samples from three additional dates in 2000 (May 25, June 9, June 22) and seven from 2002 (May 27, May 30, June 3, June 10, June 17, June 24, July 1), were selected. The third set of microarray experiment (a detailed weather study) contains samples from August 7 until August 16 from both 2003 and 2004. More detailed information about the samples can be found in UPSC-BASE [15]. Temperature, precipitation, irradiance for the detailed spring and weather experiment are shown in Figure 6.

### Labelling and hybridization

A common reference experimental design was used for most samples. The RNA for the common reference was a pooled mixture of 40 RNA preparations from the year 2000 [8]. RNA was prepared according to previously published methods [48,49] with the small modifications [50]. RNA was reverse transcribed into aminoallyl-labelled cDNA, using 20 *μ*g total RNA primed with 5 *μ*g oligo-dT primer, and Superscript II reverse transcriptase (Invitrogen, Carlsbad, CA, USA). The cDNA was then purified using Microcon 30 (Millipore, Bedford, MA, USA) concentrators, eluted in H_2_O and dried in a Speed-vac centrifugal evaporator.

For the first experiments (whole season), all samples were hybridized manually against the common reference in four replicates without dye-swap using the POP1 slides described by [12]. The samples were re-suspended in 10 *μ*L of 0.1 M NaHCO_3 _in which the cDNA was coupled to Cy-3- or Cy-5-esters (Amersham Bioscience, Little Chalfont, UK) for 3 h. The labeled samples were mixed and 7.5 *μ*L of 4 M hydroxylamine was added to prevent cross-coupling. The mixed samples were purified using QIAquick columns (Qiagen, Valencia, CA, USA) and then dried in a Speed-vac centrifugal evaporator. A 30 *μ*L portion of hybridization buffer (0.5% SDS, 5× SSC, 5× Denhart's solution and 50% formamide) was added to each slide, and the slides were then incubated for 45 min at 42°C in a water bath. Each dried sample was re-suspended in 25 *μ*L hybridization buffer together with 3 *μ*L oligo-dA (10 *μ*g *μ*L-1) and 0.4 *μ*L tRNA (25 *μ*g *μ*L-1), then applied to the slide surface. The slides were hybridized for 16 h at 42°C and washed with three solutions (1× SSC, 0.2% SDS, followed by 0.1× SSC, 0.2% SDS and finally 0.1× SSC). After washing the slides were dried under a stream of nitrogen gas.

For the second set of experiments (the detailed spring study), samples were hybridized against the common reference using the POP2 arrays [51,52] in an automated slide processor (ASP; Amersham Bioscience, Little Chalfont, UK) with dye-swaps. In addition to all clones included in the POP1 arrays, POP2 also contains clones from new cDNA libraries. The samples were re-suspended in 10 *μ*L of 0.1 M NaHCO_3_, pH 9, and mixed with the appropriate Cy-dye, dissolved in 10 *μ*L DMSO. The samples were coupled for 120 minutes and cleaned using a Cyscribe GFX purification kit (Amersham Bioscience, Little Chalfont, UK) according to the original instructions. The cDNA was eluted in 60 *μ*L of elution buffer and then the volume was reduced in vacuo to 41 *μ*L. Appropriate incorporated samples were pooled and mixed with 45 *μ*L SSC (20×), 45 *μ*L formamide (100%), 4 *μ*L SDS (10%), 1 *μ*L tRNA (10 *μ*g *μ*L-1) and 3 *μ*L oligo-dA (25 *μ*g *μ*L-1). The samples were denatured at 95°C for 3 min before injecting them into ASP chambers, which were placed in the ASP. Here, the slides were exposed to pre-hybridization buffer (5× SSC, 50% formamide, 2.5× Denhart's solution, 0.1% BSA) and the samples then were injected using a syringe. The slides were hybridized for 14–16 h at 42°C then washed with three buffers of sequentially decreasing concentration (1× SSC, 0.05% SDS followed by 0.3× SSC and finally 0.05× SSC). Detailed information about the parameter settings in the ASP can be sent on request. The third set of experiment (weather study) was hybridized in a loop experiment using the POP2 arrays as described for the second set of experiment.

Slides were scanned 4–5 times with predetermined increasing laser power and phototube multiplier (PMT) settings using a Scanarray scanner (PerkinElmer AB, Sweden). The resulting images were analyzed in Genepix 5.0 (Axon Instruments, CA, USA). All TIFF images were processed with composite pixel intensity (CPI) settings set to find circular features and resize the features within 80–150% of default size (100 *μ*m)and composite pixel intensity threshold set to 300 during alignment. Weak spots were automatically marked as not found. The extracted data were stored as results files containing raw data and various statistical measurements. When necessary, the POP1 subset of samples from the year 2000 in the POP2 arrays were extracted and analyzed together with the original POP1 samples from year 2000. For the first set of microarray slides some slides were discarded due to uneven hybridisation. Numbers of kept hybridizations are indicated in parenthesis, June 1(4), June 15(2), June 29(3), July 6(3), July 18(4), July 27(4), August 3(4), August 11(4), August 18(3), August 29(4), September 12(2).

The raw data are stored in the public microarray poplar database UPSC-BASE [15] in which the first (whole season), second (detailed spring) and third (weather) have been assigned the codes UMA-0001 UMA-0032 and UMA-0078, respectively. The different scan levels for each slide were merged with Restricted Linear Scaling (RLS) [53] followed by step-wise normalization [54] before further analysis.

### Linear models

Gene-wise linear models were used to analyze the gene expression values for each sampling date. The linear model is described by *Y *= X * *β *+ *ε*, where *Y *is a vector of log-ratios from different slides, *X *is the design matrix, *β *is the vector of parameters and *α *is the error. For the third set of experiment (weather study) an insilico average was calculated to allow comparison over the time [55]. The models were calculated using functions from the package Linear Models for Microarray Data (LIMMA) [29] in the statistical software R [56] implemented in UPSC-BASE.

### Principal component analysis

Principal component analysis (PCA) is an unsupervised projection method used to extract systematic trends from large data tables [57]. Data sets containing possibly several thousand of features (e.g. expression levels of genes, protein abundances, etc.) can be reduced to a handful of principal components characterizing the strongest systematic effects according to the variance in the data. Each of these principal components (generally referred to as latent variables [58] describe independent effects in the data, which relate the samples by means of score vectors and the corresponding features using the loading vectors. PCA is most typically utilized for exploratory analysis purposes; to identify trends, clusters or outlying samples where this cannot be performed for each sample individually. In the present case, PCA was performed on the weather and microarray data using the SIMCA-P 11.5 software (Umetrics AB, Ume aa, Sweden). All variables were mean-centred and scaled to unit variance prior to weather analysis, which implies subtracting the mean value from each weather parameter and dividing it by its standard deviation. The microarray data were only mean centred. The number of principal components in the PCA model was selected according to cross-validation [see Additional file 8 for details]

### Orthogonal projections to latent structures

Orthogonal projections to latent structures (OPLS) is a supervised multivariate linear regression method [22]. In relation to the unsupervised PCA method, OPLS requires and makes use of additional background information; for instance presence of different sample groups (classes) [59] or treatment concentrations. There currently exists a multitude of supervised linear regression methods in the literature [60].

OPLS was used to predict gene expression levels based sunlight, temperature, humidity, wind and precipitation parameters on the time of sampling and earlier. To avoid the problem of over fitting, cross-validation [24] was performed to find an OPLS model with good generalization properties. Based on properties of the cross-validated OPLS model, microarray elements that were reliably predicted from the weather parameters were subsequently identified, suggesting that such elements are influenced by or directly regulated from the weather. OPLS modelling was performed using the statistical software R [56] based on in-house produced code. The microarray data set was mean-centred for each microarray element whereas weather parameters were both mean-centred and scaled to unit variance prior to analysis. OPLS model parameters were selected according to cross-validation [see Additional file 8 for details].

### Identification of microarray elements affected by weather

The ability of the OPLS model to approximate the expression profile of a microarray elements based only on the weather parameters will be denoted the predicted variation for that particular element. The predicted variation ranges from negative infinity to one, where a high positive value indicates a reliable prediction and vice versa. Such a strategy for identification of reliably predicted features has been employed previously in a different context [61]. The predicted variation estimates were subsequently converted to conventional p-values based on a permutation strategy, where the link between weather parameters and expression levels has been disrupted. This procedure was repeated numerous times in order to estimate what degree of predicted variation that could be expected by chance from a data table with equal properties [62]. The estimated p-values were subsequently corrected for multiple testing inflation using step-wise false discovery rate correction [63]. False discovery rate adjusted p-values (FDR) were called significant if FDR < 0.05, rendering a total of 199 significant microarray elements. See Figure 4 for an overview of the microarray elements and the relation to the weather parameters.

### Comparing array elements effected by environmental vs. developmental control

For the weather dataset the *in silico *reference was calculated and array elements having a positive B-statistic value for each date were merged into a unique environmental gene set. For the detailed spring dataset the B-statistic were calculated against the seasonal reference and the two lists of array elements were compared.

### Local modelling to highlight weather-gene links

The global OPLS model describes a many-to-many relationship between microarray elements and weather parameters. This is beneficial from a multivariate perspective but it may be non-trivial to directly pinpoint the weather parameters that are predominantly influential for one particular microarray element. For this purpose, we constructed local OPLS models for each microarray element where weather parameters were used to sequentially predict one microarray element at a time. From the predictive loadings of each local model an approximation of the influence of weather parameters for each individual microarray element is achieved. This is shown in Figure 5 for a set of selected microarray elements.

### Analysis of functional classes

To assess changes related to interesting biological themes systematically we used a version of the GOstats Bioconductor package [16], modified for applications with our poplar array and implemented in UPSC-BASE [15] using information obtained from the PopulusDB [51,64] and the *Arabidopsis *Information Resource (TAIR) [65]. Functional classes for the closest *Arabidopsis *orthologue to each of the *Populus *genes were collected from Gene Ontology (GO) [66] definitions. The GOstats inputs were lists of significant genes [67]. The output was parsed and used to follow time trends in the biological themes and classes with p-values less than 0.05 were regarded as important. The results were summarized and plotted on a GO graph structure. Only categories over-represented at one or more time point and their parents were used in the graph.

### Temperature sum

Temperature sums were calculated as the accumulated daily mean temperature above the threshold value 1°C [36]:

(1) $$TS(t)=\sum_{t_{0}}^{t}TS(t)-T_{b}$$

Where TS is the temperature sum on day t, T(t) is the daily mean temperature (°C) on day t, *T*_*b *_is the threshold temperature (1°C) and t_0 _is the starting date of the temperature sum accumulation, here January 1st [68].

## Authors' contributions

AS collected leaf samples, performed microarray lab work for the POP2 dataset, analyzed all microarray data and drafted the manuscript. KW collected leaf samples and generated the initial POP1 seasonal microarray dataset. MB analyzed the weather to gene correlations and provided expertise primary regarding OPLS. SJ conceived the study and supervised the project together with JT. All authors read and approved the manuscript.

## Supplementary Material

Additional file 1 **Description of weather parameters**. Description of the abbreviated weather parameters used in the figures. Click here for file

Additional file 2 **Data file containing weather parameters**. The data used for the weather calculations. Click here for file

Additional file 3 **Graphical representation of the weather during the growing season in the year 2000**. The first two principal components in the PCA analysis of the weather parameters are shown. White circles depict calendar dates during the growing season (May 15 to October 15), grey circles show the eleven dates selected for the whole season experiment. The grey circle to the right marks the controlled outlier. Click here for file

Additional file 4 **Gene expression in leaves of free-growing aspen during the growing season**. The first two principal components obtained from the PCA analysis of the microarray data from year 2000 are shown. Technical replicates are given the same symbol. Click here for file

Additional file 5 **Heatmap of the over-represented Gene Ontology categories in the spring samples**. The samples from the spring 2000 and 2002 are ordered according to developmental leaf age. Red squares are categories over-represented in significantly up-regulated genes and green squares are categories over-represented in significantly down-regulated genes. Divergent groups that were over-represented in both up- and down-regulated genes are plotted as yellow squares. This figure shows all significant categories of the selection showed in Figure 3. Click here for file

Additional file 6 **Significant Gene Ontology categories for genes differentially expressed during development**. List of gene ontology categories showing significant over-representation during leaf development. Click here for file

Additional file 7 **Significant Gene Ontology categories for genes differentially expressed during weather influence**. List of gene ontology categories showing significant over-representation during weather influence. Click here for file

Additional file 8 **Description of used cross-validation**. More extensive description about the cross-validation used in OPLS. Click here for file
