# Supplementary material for: Global expression profiling in leaves of free-growing aspen
Source: BMC Plant Biol. 2008 May 23;8:61. doi: 10.1186/1471-2229-8-61 (PMC2416451; doi:10.1186/1471-2229-8-61)
Supplement: Additional file 8 — Description of used cross-validation. More extensive description about the cross-validation used in OPLS. [file 1471-2229-8-61-S8.doc]

## Cross-validation and predicted variation

Cross-validation is a conceptual solution for estimating parameters (for instance variance or rank) where this cannot be done in closed form. The full data is split into *n* groups randomly, where the groups should be of (roughly) equal size. Subsequently, a model (for instance, an OPLS model) is generated from all groups but one. The excluded group is predicted from the estimated model and as the true result is known, one can calculate the prediction error (typically in the form of the sum of squared errors). This is repeated so that all groups are left out once. This procedure is commonly referred to as *n*-fold cross-validation, which is the method that has been employed for *n*=5.

Cross-validation is used primarily to estimate the generalization error or *predicted variation*. The predicted variation is frequently normalized against the total variance in the data (SS_tot_), and estimated using the formula in Equation S1.

*Q*^2^ = 1 – PRESS/SS_tot_ (Eq. S1)

One can see from Equation S1 that when the predicted variation (PRESS) decreases, which is the optimal scenario, then the *Q*^2^ value will increase. Consequently, one wants to achieve the highest possible *Q*^2^; although this value can never be higher than 1 (which occurs when PRESS is exactly zero). A *Q*^2^ value of zero, on the other hand, implies that PRESS is equal to RSS, which means that the model predicts as good as one would by guessing the mean value of each variable all the time; a poor model indeed. Consequently, *Q*^2^ > 0 implies that the model has predictive abilities and that the higher the value of *Q*^2^, the smaller the generalization error. It is also possible to estimate the value of *Q*^2^*_i_* for each feature *i* (e.g. microarray element *i*) separately, which is the methodology that has been employed here to identify microarray elements that are reliably predicted from weather parameters.

For the OPLS model employed here, the highest overall predicted variation of 11.7% was achieved for 3 predictive and one **Y**-orthogonal components (see (Trygg and Wold 2002) for a definition of these parameters). This low overall predicted variation is expected due to the obvious problems of trying to predict gene expression profiles from weather parameters where such relations may not exist. The explained variation by the model was 59.7% for the microarray data table and 76.1% for the weather parameters.

## The permutation strategy

The following permutation approach has been employed to calculate *p*-values from the predicted variations *Q*^2^*_i_*. Samples in the microarray data table were reshuffled while maintaining the order in the weather data table 1000 times so that the original relations between the data sets are decreased or eliminated. From this reshuffled data, the distribution of *Q*^2^*_i_* is estimated, which denotes the distribution one would expect from pure chance of a data set with similar characteristics (approximating a *null* distribution). Given a microarray element *i* and its predicted variation *Q*^2^*_i_*, the *p*-value was calculated based on the density of the *null* distribution and subsequently adjusted using FDR correction (Benjamini and Hochberg 1995).

## Local modeling

For the local OPLS models, one predictive and one **Y**-orthogonal component were employed throughout (see (Trygg and Wold 2002) for a definition of these parameters).

## References

Benjamini Y, Hochberg Y (1995) Controlling the false discovery rate: a practical and powerful approach to multiple testing. J R Stat Soc B 57: 289-300

Trygg J, Wold S (2002) Orthogonal projections to latent structures (O-PLS). J Chemometrics 16: 119-128
